# Prevalence and pattern of emotional abuse of children in their homes; self reported experience of children in Ilorin Nigeria

**DOI:** 10.1192/j.eurpsy.2021.1890

**Published:** 2021-08-13

**Authors:** A. Oladosu, O. Abiodun, M. Tunde-Ayinmode

**Affiliations:** 1 Johnson Community Hospital, Lincolnshire Partnership Foundation Trust, Lincolnshire, United Kingdom; 2 Behavioural Sciences, University of Ilorin Teaching Hospital, Ilorin, Nigeria

**Keywords:** Child, emotional abuse, home

## Abstract

**Introduction:**

The prevalence and pattern of emotional abuse of children in Nigeria is poorly understood. Data from other parts of the world indicate it is commoplace and has enduring negative mental health impact. The current study aims to understand the phenomenon the more.

**Objectives:**

To determine the prevalence and pattern of emotional abuse of children in their homes in Nigeria

**Methods:**

Cross sectional survey of 1, 5444 secondary school students aged 11-18 years in Ilorin Nigeria using multistage random sampling technique with proportional allocation was done. Respondents completed the ICAST-CH questionnaire which covers child abuse in its several forms including emotional abuse. Prevalence of emotional abuse was computed.

**Results:**

All respondents (100%) had experienced emotional abuse at home in the last one year Table 1: Prevalnce annd pattern of emotional abuse at home among children in Ilorin Nigeria

**Table:** 

Emotional Abuse* (n=1554)	Frequency	Percentage
Threatened to hurt or kill you or threatened with evil spirits	1554	100
Screamed at	1528	98.3
Bullied by another child at home	1218	78.4
Insulted	1122	72.2
Made you feel embarrassed	574	36.9
Wished you were dead	224	14.4
Locked out of home	196	12.6
Threatened to abandon you	95	6.1

**Conclusions:**

Emotional abuse of children at home is common place in Ilorin Nigeria. It would seem important to educate parents on what emotional abuse is and its potential impact in children.

**Disclosure:**

No significant relationships.

